# The Impact of “Unseasonably” Warm Spring Temperatures on Acute Myocardial Infarction Hospital Admissions in Melbourne, Australia: A City with a Temperate Climate

**DOI:** 10.1155/2014/483785

**Published:** 2014-06-04

**Authors:** Margaret Loughnan, Nigel Tapper, Terence Loughnan

**Affiliations:** ^1^CRC for Water Sensitive Cities, School of Geography and Environmental Science, Monash University, Wellington Road, Clayton, VIC 3800, Australia; ^2^School of Medicine Nursing and Health Science, Monash University, Wellington Road, Clayton, VIC 3800, Australia

## Abstract

The effects of extreme temperatures on human health have been well described. However, the adverse health effects of warm weather that occurs outside the summer period have had little attention. We used daily anomalous AMI morbidity and daily anomalous temperature to determine the impact of “unseasonable” temperature on human health. The “unseasonably” warm weather was attributed to a slow moving high pressure system to the east of Melbourne. No morbidity displacement was noted during either of these periods suggesting that morbidity due to “unseasonable” temperatures is avoidable. An increase in warmer weather during the cooler months of spring may result in increased morbidity, and an alert system based on summer thresholds may not be appropriate for early season heat health warnings. A straightforward alert system based on calculating anomalous temperature from daily weather forecasts may reduce the public health impact of “unseasonably” warm weather.

## 1. Introduction

The relationship between heat waves and increased mortality and morbidity has been well described [1–4]. Similarly, an increase in population mortality and morbidity resulting from cold weather has also been well documented [5–10]. Thus, the adverse health effect of excessively hot weather during the warm season and of cold weather during the cool season is well known. However, what is unclear is the possible effect of anomalously warm temperatures that occur at other times of the year, that is, “unseasonably” warm weather during normally cool or mild seasons.

Understanding the adverse health implications of unseasonably warm weather throughout the year may be important for predicting the impact of climate change. If milder winters in the future also feature “unseasonably” warm temperatures could this result in an adverse health response, possibly offsetting any decreased mortality/morbidity that might be expected from the warmer winters?

Ebi et al. [11] evaluated the effects of temperature increases and decreases in hospital admissions for cardiovascular diseases in California, US. Temperature changes were associated with a 6–13% change in CVD admissions [11]. Saez et al. [12] found that unusual periods of weather associated with increased temperature and humidity increased mortality independent of the V shaped temperature mortality relationship previously described for Barcelona, Spain [12]. This occurrence of an unusual period of increased temperature for at least 3 consecutive days resulted in an increase in total daily mortality of 2% on average and 2.6% in those aged 65 years and older (1.7% and 2% in warm season, resp.). In persons aged 65 years and older, cardiovascular mortality rose by 4.6% and respiratory mortality by 21.6% (4.2% and 13.2% in warm season, resp.) [12].

In this study we describe the health impacts of two periods of anomalously high temperatures that occurred during spring, in Melbourne, Australia, a city with a temperate climate and usually mild springtime temperatures. Heat waves in Melbourne during summer can lead to excess mortality [13, 14] and morbidity [7]. However previous studies have not examined the health impacts of anomalously warm periods during the milder times of the year. The aim of this study was to use case studies to describe the synoptic weather patterns associated with anomalously warm episodes during the spring months of the year and the adverse health impacts of high temperatures in a cohort of older men. Although the spring months described in this study were much warmer than is usual for winter and spring, they were still typically cooler than heat wave events in summer.

## 2. Methods

Melbourne is Australia's second largest city. The central business district (CBD) and suburbs comprise the statistical district (SD) of Melbourne with an estimated resident population (2001 census) of 3,488,750 persons [15].

### 2.1. Health Measure

The morbidity measure used in this analysis was the daily number of hospital admissions for an acute myocardial infarction (AMI) in Melbourne, Australia. Heart disease is the largest cause of morbidity in Australia [16] and AMI sufferers are routinely admitted to hospital so accurate data on AMI occurrences are available [8]. The study population consisted of all subjects hospitalised in the 37 hospitals in the statistical district (SD) of Melbourne during a 12-year period from 31/12/1993 to 31/12/2004 who were aged 35 years and older, were resident in the SD of Melbourne, and had a principal diagnosis of AMI. Time-series analysis was used to examine the changes in monthly AMI admissions and determine if a seasonal pattern in AMI morbidity existed in Melbourne. The dependent variable of interest was mean monthly admissions rate. Daily admission rates adjusted yearly for population change were calculated. Mean monthly admission rates were calculated from the daily data for each month of each year. The independent variable was a time component of monthly intervals for 11 years. Time-series analysis was performed using SPSS [17] to produce sequence charts to display seasonal cycles and long-term trends in the data. General linear models were used to determine seasonal patterns and trends over time. Mean monthly admission rates were calculated to describe the seasonal pattern in AMI admissions. To examine the joint effects of age group, sex, and month on AMI admissions several groups were examined; AMI admissions were initially divided by sex (male, female) and into two age groups (35–54 years and 55 years and older) and finally into four groups consisting of age∗sex (males 35–54, females 35–54, males 55 years and older, and females 55 years and older). Seasonal patterns for all groups were examined. One-way analysis of variance (ANOVA) was used to determine if there were statistically significant differences between groups of cases during the October case studies.

The International Classification of Disease (ICD) version 9 codes 410.0–410.9 and ICD-10 codes I21.0–I21.9 were used to identify AMI admissions. Data were supplied by the Department of Human Services (DHS) from the Victorian Admitted Episode Dataset (VAED) and included all five Melbourne hospital regions, date of admission to hospital, principal diagnosis, and Statistical Local Area in which each patient resided. The VAED is audited internally every two years and has a published 0.9% error rate [15]. Ethics approval for this study was granted by the Monash University Standing Committee on Ethical Research involving Humans.

### 2.2. Weather Data

To determine the effect of unseasonably warm weather the daily maximum and minimum temperature for Melbourne Regional Office weather station were obtained for the study period from 1993 to 2004. Years, months, and days were aligned and a daily average maximum and minimum temperature were calculated. This was used as the daily expected temperature from which the daily temperature anomaly (°C) was calculated for each day in the study period. This was done for daily maximum and daily minimum temperature from 1993 to 2004. Only one weather station was used as previous work had shown a strong correlation between Melbourne Regional Office (in the central business district) and surrounding suburban weather stations for daily maximum and minimum temperatures [7, 8]. Cases with temperature anomalies greater than 1°C were investigated; that is, only unseasonably warmer conditions were examined here; Table 1 shows the monthly average temperature for each month. Weather station data and relevant synoptic charts for the case studies presented below were provided by the Australian Bureau of Meteorology.

## 3. Results

Examination of the times-series using mean monthly admissions to hospital revealed a seasonal cycle and a positive long-term trend in the data. To explain the positive trend over time standard regression analysis using a General Linear Model (univariate analysis) [17] found a pattern in residuals. The pattern indicated no significant trend in younger (<55 years) male and female admissions but a significant positive trend for older male admissions and older female admissions (*r* ^2^ = 0.44, *P* = 0.047).

**Figure 1:**
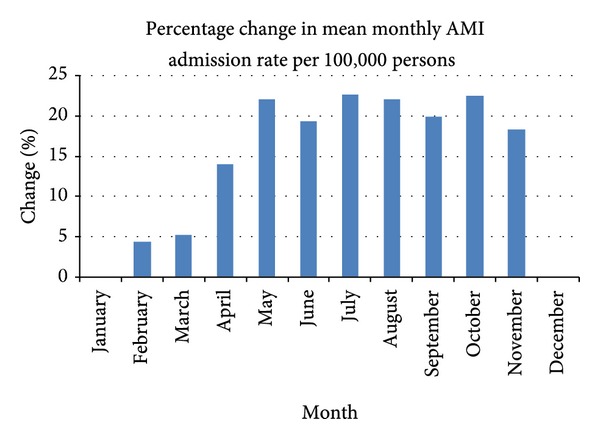
Percentage difference in mean monthly admission rate relative to base month December.

The monthly percentage change in mean AMI admissions across the years shown in Figure 1 clearly demonstrates a seasonal pattern with an increase in the admission rate from April to November. The peak month is July with a 22.7% increase over the base month of December. This pattern of increased admission rates is broader than the traditional winter months from June to August.

**Figure 2:**
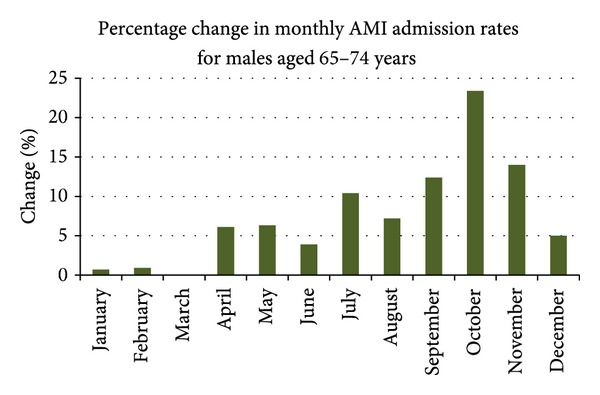
Monthly percentage differences for males aged 65–74 years.

Further examination of seasonal patterns by age and sex showed an interesting pattern for males aged 65–74 years, with a large increase in October (Spring; see Figure 2).

Data for males aged 65–74 years in each October month were extracted and detailed examination of the weather during October indicated that weather was highly variable with both anomalously warm and cool temperatures occurring. Further analysis revealed two episodes of much warmer than expected weather that were associated with increased AMI admissions. The first episode was in October 1994 and the second was in October 2004. These episodes are presented as two case studies.

### 3.1. Case Studies

**Figure 3:**
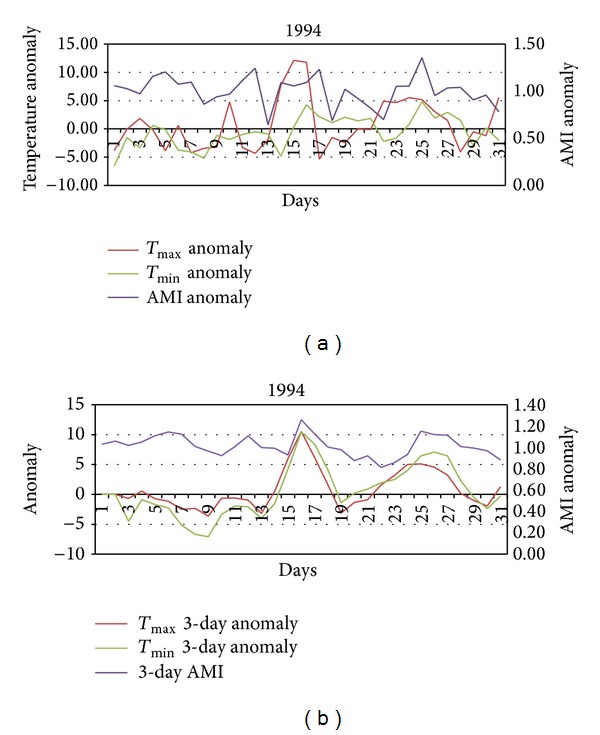
(a) Daily temperature and AMI anomalies during October 1994. (b) 3-day averages of daily temperature and AMI anomalies during October 1994.

**Figure 4:**
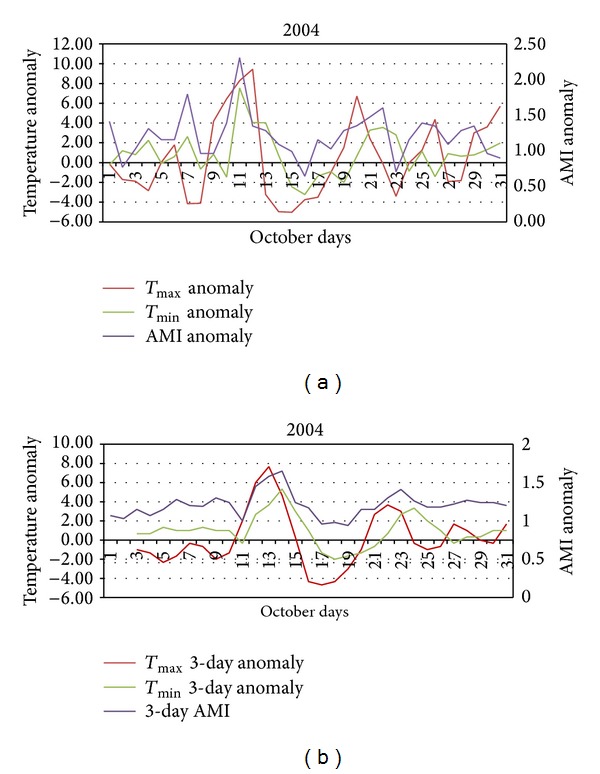
(a) Daily temperature and AMI anomalies during October 2004. (b) 3-day averages of daily temperature and AMI anomalies during October 2004.

The daily excess AMI admissions and weather anomaly data for October 1994 and 2004 were extracted from the data set. These data were graphed to demonstrate the relationship between daily maximum and minimum temperature and AMI admissions (see Figures 3(a), 3(b), 4(a), and 4(b)).

#### 3.1.1. Case Study 1994

The first period of unseasonably warm temperature between the 15th and 17th of October was between 6°C and 10.5°C warmer than expected for that time of year (Figure 3(a)). This increase in temperature was associated with an increase in both AMI admissions. AMI admissions increased quickly on the 15th and then fell on 16th and remained low thereafter. The second period of unseasonably warm weather occurred from the 23rd to the 27th of October. Overall, the daily AMI admissions appear to be less clearly related to elevated maximum temperatures and more clearly related to elevated minimum temperatures. There is a well described lag of 1–3 days [11, 12, 18] in coronary artery disease (CAD) morbidity following hot weather. When the 3-day average AMI was examined with 3-day average temperature anomalies a clear pattern emerges (Figure 3(b)). An AMI anomaly greater than 1 suggests more than expected AMI admissions to hospital. During both the aforementioned warmer periods the AMI anomaly ranges between 1.11 and 1.26. The results of the ANOVA indicate that statistically significant differences exist for 3-day average AMI between days that are warmer than average (*T*_max⁡_) and other October days in 1994 (*F* = 4.378 and *P* = 0.04).

#### 3.1.2. Case Study 2004

The data for October 2004 are shown in Figures 4(a) and 4(b). Again using a 3-day average has shown a clearer pattern. The initial period of unseasonable weather was between 9th and the 13th of October. This period was between 1°C and 9.5°C warmer than expected and this unseasonably warm period was associated with an increase in AMI admissions to hospital. A second period of increased maximum and minimum temperature between the 19th and 22nd of October was also associated with an increase in AMI admissions albeit a weaker response. Subsequent warm days on the 26th and 29–31st of October also show an increase in AMI admissions. Overall, unseasonably warmer than expected temperatures in October 2004 were associated with an increase in AMI admissions to hospital. AMI anomalies for periods of hotter than expected weather range from 1.65, from the 9th to 13th, to 1.41, from the 19 to 22nd. The association appears to be stronger for minimum temperature anomalies as shown by the small increase in AMI admissions at the beginning of the month when overnight temperatures were warmer than expected but daytime temperatures were cooler than expected. Warmer than expected overnight temperatures prolong heat exposure and may heighten the health response in susceptible people. The results of the ANOVA indicate that statistically significant differences exist for 3-day average AMI between nights that are warmer than average (*T*_min⁡_) and other October nights in 2004 (*F* = 8.51 and *P* = 0.007).

#### 3.1.3. Synoptic Charts for the October 1994 and 2004 Events

**Figure 5:**
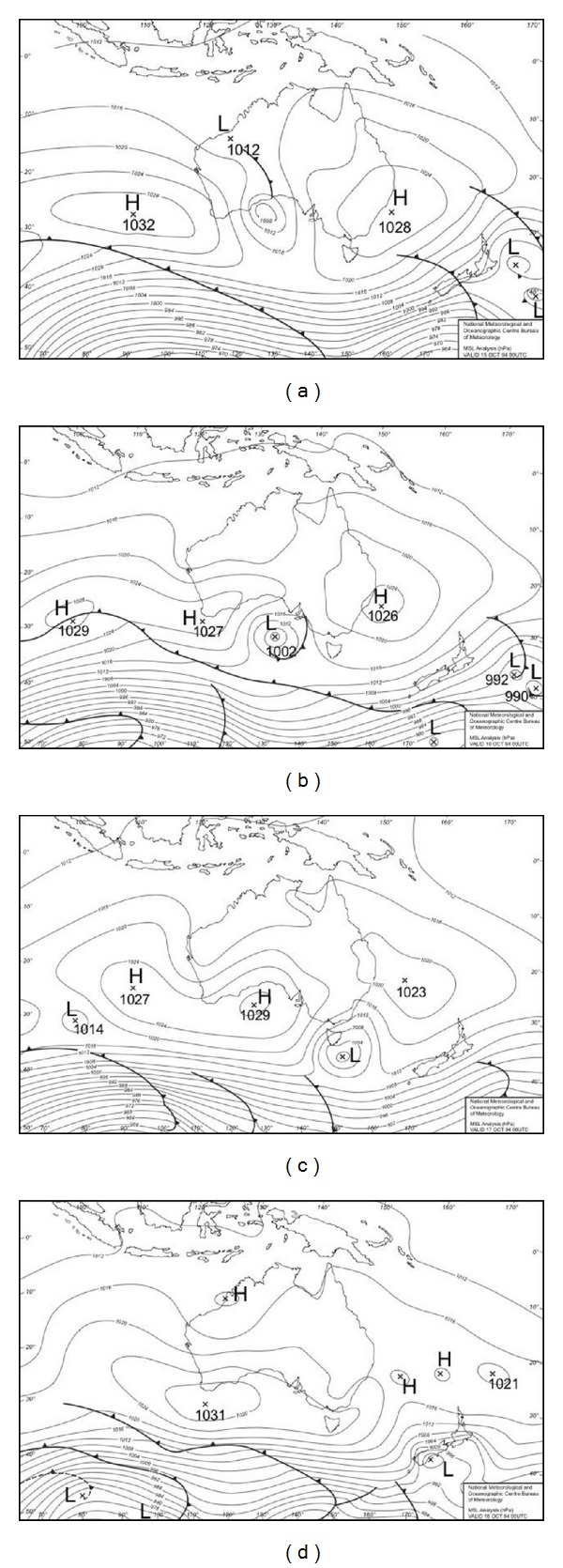
Synoptic charts showing mean sea level pressure (MSLP) analyses for the Australian region for the period 15–18 October 1994 (a–d respectively). The locations of high and low pressure and frontal systems are shown on the charts. Data are from the Australian Bureau of Meteorology.

**Figure 6:**
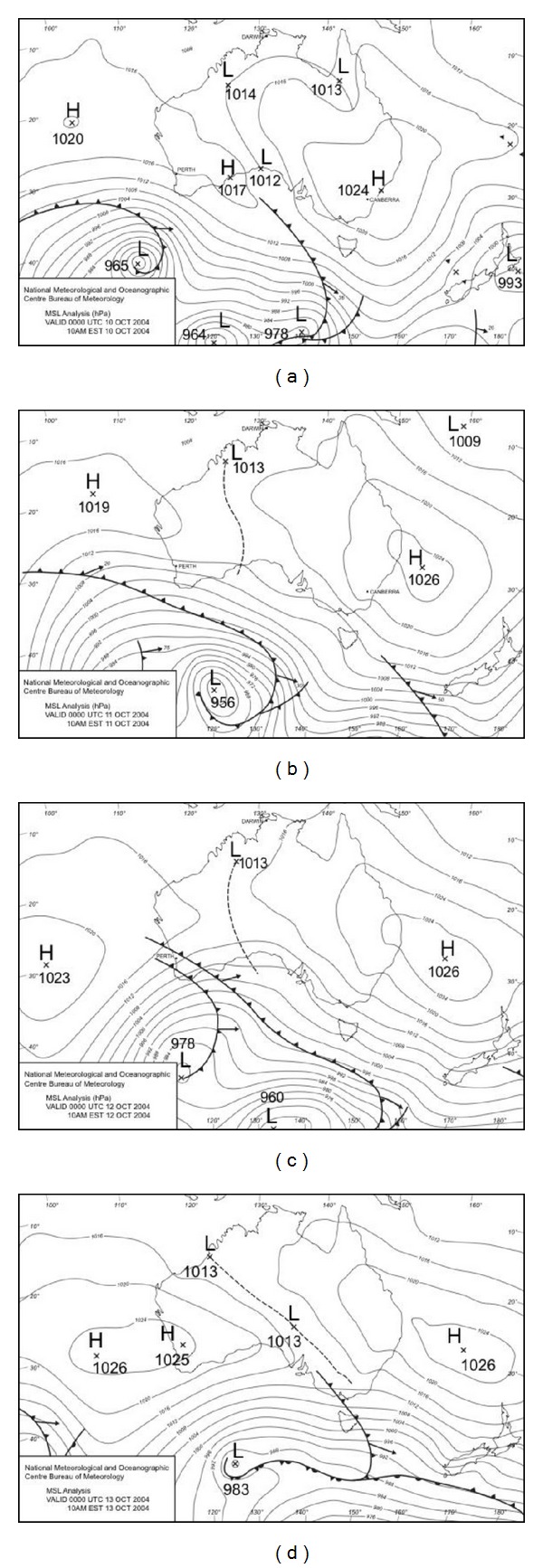
Synoptic charts showing mean sea level pressure (MSLP) analyses for the Australian region for the period 10–13 October 2004 (a–d respectively). The locations of high and low pressure and frontal systems are shown on the charts. Data are from the Australian Bureau of Meteorology.

In order to better understand the “heat anomalies” during October synoptic weather patterns were examined. In Melbourne warm weather is driven by high pressure systems that bring warm continental air from the inland arid regions down over the city. Synoptic weather charts showing mean sea level pressure (MSLP) distributions and the presence of frontal systems in the Australian region relating to relevant periods of these two months are shown in Figures 5 and 6. The periods for which charts are provided are the 15th to 18th of October 1994 and the 10th to 13th of October 2004. The common element for both of these events is a large slow moving or stationary high pressure system located off the east coast of Australia near Brisbane, drawing warm northerly airflow over Melbourne from the interior of the continent (air circulation is anticlockwise around a high pressure system in the Southern Hemisphere).

During the 1994 event daytime temperatures of close to 30°C on the 16th were ameliorated as a low pressure system moved to the southeast of Melbourne on the 17th, drawing cooler westerly, then southwesterly, flow over the city. The 2004 event was characterised by a more persistent northerly flow that maintained day and night temperatures well above normal (along with AMI admissions) for several days.

## 4. Discussion

Using a simple approach to identify “unseasonable” temperatures and anomalous AMI morbidity, this study demonstrates that periods of “unseasonable” temperature are associated with increased AMI admissions to hospital Melbourne. This association occurs independently of the “cool season” and the “warm season” relationships between temperature and mortality and morbidity noted in Melbourne [7, 14] and elsewhere [9, 19–22]. Finding that even in cooler months unseasonably warm weather can lead to excess AMI morbidity is of concern, given the expectation of milder cool season temperatures, increased heat events, and increased climate variability in many regions in response to global climate change. The case studies suggest that even in cooler seasons anomalously warm temperatures may lead to excess AMI morbidity. The Australian Bureau of Meteorology provides synoptic forecasts for 4-day periods; therefore, public health awareness campaigns could easily alert susceptible individuals to the possible risk associated with warmer than expected temperatures during spring. Reducing exposure to ambient heat in spring and minimising exertion in warmer weather would be an important health message.

These results have interesting implications for predicted climate change in two ways. Firstly, the cool season temperatures are expected to become milder [23]. If this results in more episodes of anomalously warm temperatures especially milder overnight temperatures during the cooler spring months, this may result in increased AMI morbidity from heat exposure due to lack of acclimatization. Several studies have suggested that heat related mortality and morbidity is greater during the heat events that occur early in the warm season period [22, 24]. These case studies from Melbourne suggest that a single threshold may not be comprehensive enough to predict the impacts of hot weather on health and consideration of relatively “hot” days during spring is important. Secondly, some studies [25, 26] suggest that during the cool season “warming” may result in decreased mortality and morbidity. The results presented here suggest that if cool season “warming” leads to more periods of unseasonably warmer days this may offset the predicted expected decrease in mortality and morbidity in the cool seasons.

A seasonal increased admission rate from May to November is most evident in the 65–74 years, and the 75 years and older groups; this is skewed towards the spring months of September, October, and November. The explanation as to why susceptible people have AMI in a hot spell in cooler months is most likely to be multifactorial rather than a single cause. Many retired Melbournians holiday in the northern states during the winter period and this spring peak may be related to their return to Melbourne. After several months of holiday in a warm climate and 4–7 days of driving, returning home to an unkempt garden and home may incur physical exertion enough to trigger an MI. During colder months people tend to become less active and their fitness level can be expected to decline. A sudden increase of activity above usual levels results in significant increases in myocardial oxygen demands, and in susceptible individuals when the myocardial oxygen requirement exceeds myocardial oxygen supply the result is an acute myocardial infarction. Thus people who suffer from significant ischemic heart disease, who have become sedentary over winter, and who decide to engage in physical exertion because of the warmer weather will be at risk of inducing an AMI. This is not seen in persons who are fitter or who gradually increase activity levels. Public health messages about maintaining fitness throughout the year are of importance. Additionally, a possible contributing factor is that elderly people have a lower thirst response to dehydration and hotter weather with increased exertion results in increased sweating, which if coupled with decreased fluid intake results in dehydration. This in turn leads to a drop in circulating blood volume, a decrease in blood pressure with orthostatic hypotension (low blood pressure when upright) which reduces blood flow to the heart potentially leading to an AMI. This series of events combine both behavioural and physiological responses. It is intuitive that alterations of behaviour patterns can be achieved far quicker than alterations in physiological response. The key messages presented here are to use readily available information from the Bureau of Meteorology to issue heat health advisories during spring and to reiterate the importance of maintaining fitness, avoiding exposure during warm weather, and to maintain hydration when working or exercising, even during the milder months.

Of note with the pattern in 1994, the graph shows that higher temperatures are associated with an increased AMI rate. From around 1999 the accepted criteria for making a diagnosis of an AMI have altered from a history and symptoms consistent with AMI, recognised diagnostic changes on the electrocardiogram (ECG), and a blood test for heart damage. Since 1999 there has been an additional blood test, Troponin. These enzymes are a chemical contained within heart muscle cells and their presence in the blood indicates death of cardiac muscle cells. This Troponin rise is felt to be more sensitive and indicative of heart cell damage that may not result in changes that met earlier criteria. In a Scottish Hospital the introduction of the new criteria (Troponin assay) resulted in an increase of admissions for AMI of 58%. These additional patients were identified as being older with a higher proportion of women with poorer survival [27]. The introduction of Troponin tests occurred around about the same time in Melbourne and the two case studies examined here may therefore represent different diagnostic criteria. This may explain in part the greater increase in AMI admissions per increase in warmer temperatures in 2004.

With specific reference to AMI admissions to hospital, one factor that should also be noted is that there are well-documented seasonal changes in biophysiological cardiac risk factors that result in hypercoagulability [28–30]. These may well extend into the spring period, increasing the risk for some people. Another element that may influence the effect of unseasonable temperatures during spring is the size of the susceptible pool of persons at risk of death or hospitalisation during hot weather. For example, if an influenza epidemic was recorded in the preceding cool season then the susceptible pool would be smaller than expected. This may in part explain the time difference in these case studies.

A warm season threshold temperature for Melbourne of 30°C mean daily temperature has been determined for AMI admissions to hospital [7]. Based on the case studies examined here, this particular threshold does not apply to unseasonably warm days outside of the summer season. During the spring in Melbourne weather patterns are changeable and unseasonably warm weather can occur and can be associated with increased AMI morbidity despite not reaching this threshold 30°C temperature.

As shown in Figures 3 and 4 the anomalously high springtime temperatures associated with increased AMI admissions are linked with particular synoptic situations. Melbourne is a warm temperate city on the southern margins of a large, dry subtropical to tropical continent.

During winter the Australian continent is relatively cool, but it warms rapidly during the austral spring (September and October) and this is responsible for particularly active frontal systems at that time of year [31, pp 238]. As they move towards Melbourne these active frontal systems draw warm to hot air from the centre of the continent, pushing up day and night temperatures; this subtropical air mass is then replaced by much cooler southern maritime air as the front moves to the east of Melbourne. A particular feature of atmospheric circulation in the region is the relatively high tendency for “blocking” situations to develop near New Zealand [31, pp 202–204] causing high pressure systems to become stagnant or to remain slow moving just to the east of Australia. Such a situation was responsible for the relatively persistent October 2004 event. One area of concern is that climate change has the potential to influence the location and intensity of blocking in the Australasian region [32]. In addition, and of some concern here, recent modelling of future synoptic patterns for Victoria and their potential air quality impacts [33] has indicated a substantial increase in the frequency of occurrence of the synoptic mode (Mode 3 in that report) that most closely resembles the synoptic pattern shown here for the 2004 event. Mode 3 is predicted to increase in frequency of occurrence to 23% of all synoptic modes by the end of the 21st century, becoming the most common synoptic mode for Victoria. This represents an increase in frequency of occurrence for that mode of 14% over the 1961–2000 baseline frequency.

Poor air quality is recognised as a confounder of heat-health relationships. Analysis of air quality and AMI using this dataset has been undertaken previously and although an association between air quality rated as poor to very poor and AMI was identified. The association occurred during the winter period and was associated with cold overnight temperatures. Furthermore this relationship was not statistically significant [8]. Air quality readings for Melbourne were assessed [34] and were rated from good to very good for the days identified in these case studies; therefore, no further examination of the link between air quality and AMI was conducted here.

With growing numbers of elderly people worldwide, especially in larger urban centres, the demands on public health care systems will increase dramatically. The need for strategies to limit the adverse effects of heat exposure on this population is of vital importance. This work presents the opportunity to develop simple thresholds identifying anomalous springtime temperatures associated with increased risks of mortality or morbidity. This can be achieved using readily available Australian Bureau of Meteorology forecasts for days that are forecast to be >4°C above the October average daily maximum temperature of 19.9°C. Appropriate thresholds that incorporate unseasonable weather in spring will provide better protection for vulnerable members of society such as the elderly.

An interesting point to note is that there appears to be slight morbidity displacement associated with the unseasonable weather in both case studies. A peak in AMI admissions during the warmer periods is followed by a small trough in each figure. The morbidity tends to return to values close to those expected for that time of the year quickly. Thereby suggesting that the warmer weather is precipitating AMI in some cases and public health advisory systems would be helpful to mitigate the impact on people with CAD.

The main limitation of this study is that information relating to a person's general health, place of AMI, and activity was not available. Therefore we cannot verify the extent of thermal exposure experienced by persons during periods of “unseasonable” temperature.

The results presented herein may be useful to predict the impact of “unseasonable” temperature on hospital coronary care units in particular. This may be of heightened importance during the cool season when the “seasonal” increase in AMI admissions already results in an increased demand for hospital beds.

It is possible to predict periods of unseasonably warm weather using readily available Bureau of Meteorology forecasts. Public health education is an important feature of health promotion and understanding the environmental risks associated with weather and CAD would better prepare people to avoid heat exposure even during the “milder” months in spring. Future research should include a broader diagnostic base for morbidity to determine the influence of “unseasonable” weather on population health. Additionally a larger sample and demographic analysis would provide information relevant to identifying the most vulnerable groups within the Melbourne population.

## Figures and Tables

**Table 1:** Average monthly temperatures Melbourne Australia during study period 1993–2004.

	*T*_max⁡_	*T*_min⁡_
Jan	26.8	16.2
Feb	27.8	17.5
March	23.8	15.1
Apr	20.9	11.7
May	17.6	9.6
June	14.7	8.9
July	13.6	7.9
Aug	14.9	7.6
Sept	17.8	9.9
Oct	19.9	11.4
Nov	22.8	13.1
Dec	24.1	14.8
